# Innate Immune Responses of Skin Mucosa in Common Carp (*Cyprinus Carpio*) Fed a Diet Supplemented with Galactooligosaccharides

**DOI:** 10.3390/ani10030438

**Published:** 2020-03-05

**Authors:** Elzbieta Pietrzak, Jan Mazurkiewicz, Anna Slawinska

**Affiliations:** 1 Department of Animal Biotechnology and Genetics, UTP University of Science and Technology, Mazowiecka 28, 85-084 Bydgoszcz, Poland; elzbieta.pietrzak@utp.edu.pl; 2 Division of Inland Fisheries and Aquaculture, Institute of Zoology Wojska Polskiego 71c, Poznan University of Life Sciences, 60-625 Poznań, Poland; jan.mazurkiewicz@up.poznan.pl

**Keywords:** fish, GOS, prebiotic, mucosal immunity, reference genes, skin-associated lymphoid tissue

## Abstract

**Simple Summary:**

The skin mucosa in fish is equipped with innate immune mechanisms, which constitute the first line of defense against potentially harmful factors in the aquatic environment. Prebiotics, such as galactooligosaccharides (GOS), mediate modulation of the immune responses. Dietary prebiotics promote the development of intestinal microbiota, which interacts with the immune system. In this study, we analyzed the effects of the GOS prebiotic applied as a feed additive on molecular responses in the skin mucosa of the common carp. The genes analyzed encode various proteins associated with the innate immune response in skin mucosa in fish, such as mucin secretion, antimicrobial peptides, and enzymes. Modulated expression of those genes at the mRNA level regulates the defense mechanisms in the skin mucosa. In this study, supplementation with GOS increased the mRNA level of interferon and lysozyme, which are involved in fighting infection. At the same time, GOS decreased the mRNA level of CRP, which is a marker of inflammation. We conclude that supplementation with GOS modulates innate immune processes in the skin mucosa of common carp. Further studies could be focused on analyzing the effects of GOS on the microbiota composition of the skin mucosa and the mitigating effects against environmental stress.

**Abstract:**

Galactooligosaccharides (GOS) are well-known immunomodulatory prebiotics. We hypothesize that GOS supplemented in feed modulates innate immune responses in the skin-associated lymphoid tissue (SALT) of common carp. The aim of this study was to determine the impact of GOS on mRNA expression of the immune-related genes in skin mucosa. During the feeding trial, the juvenile fish (bodyweight 180 ± 5 g) were fed two types of diet for 50 days: control and supplemented with 2% GOS. At the end of the trial, a subset of fish was euthanized (n = 8). Skin mucosa was collected, and RNA was extracted. Gene expression analysis was performed with RT-qPCR to determine the mRNA abundance of the genes associated with innate immune responses in SALT, i.e., acute-phase protein (CRP), antimicrobial proteins (*His2Av* and *GGGT5L*), cytokines (*IL1β*, *IL4*, *IL8*, *IL10*, and *IFNγ*), lectin (*CLEC4M*), lyzosymes (*LyzC* and *LyzG*), mucin (*M5ACL*), peroxidase (*MPO*), proteases (*CTSB* and *CTSD*), and oxidoreductase (*TXNL*). The geometric mean of *40s s11* and *ACTB* was used to normalize the data. Relative quantification of the gene expression was calculated with ∆∆Ct. GOS upregulated *INFγ* (*p* ≤ 0.05) and *LyzG* (*p* ≤ 0.05), and downregulated *CRP* (*p* ≤ 0.01). We conclude that GOS modulates innate immune responses in the skin mucosa of common carp.

## 1. Introduction

The aquatic environment constantly challenges the water fauna with microbiological, physical, and chemical stressors (e.g., bacteria, viruses, parasites, osmotic pressure, and physical obstacles). Skin serves as the first line of defense against microorganisms and other stressors, and it is, therefore, considered a major immune organ in fish [1]. Fish skin consists of the dermis and a layer of the mucus-secreting epidermis (known as skin mucosa), covered with calcified scales [2]. The skin mucosa has high metabolic activity and unique morphology [3]. Fish skin has developed distinct mechanisms of mucosal immunity. First, it is covered by mucus that prevents pathogens from sticking to the skin surface. Second, it contains a large variety of antibacterial compounds, including proteins and enzymes, such as lysozyme and proteolytic enzymes, immunoglobulins, complement proteins, lectins, and C-reactive proteins [4,5]. Third, the dermis and epidermis contain a number of immunocompetent cells, including epithelial, mucus, club, and goblet cells, that account for the skin-associated lymphoid tissue (SALT) [6,7]. The major function of SALT is to locally recognize antigens in the skin and neutralize them with various types of innate and specific mechanisms [8]. Immunoglobulin T (IgT) plays a significant role in the immune responses mounted by SALT, which directly resembles the mechanisms of the intestinal mucosal immunity [9].

An important adaptation of the skin mucosa to the aquatic environment is the microbiota inhabiting the mucus. Skin microbiota consists mostly of the commensal bacteria, with a population of about 10^2^–10^4^ bacteria per cm^2^ of the skin [10]. Most fishes are oviparous, which means that from the moment of hatching, they are exposed to the microorganisms inhabiting water reservoirs. For this reason, a rich skin microbiota enhances the fish’s protection against environmental pathogens [11]. The known mechanisms in which skin microbiota prevents colonization of the pathogens are competitive exclusion and secretion of antimicrobial compounds [12,13]. The structure of the microbial community in the skin microbiota reflects the surrounding environment and is characterized by large interspecies diversity [14,15]. For example, 16S rRNA analysis of intestinal samples from several fish species revealed that the most abundant order in freshwater fish is *Aeromonadales*, whereas in saltwater—*Vibrionales*. It shows the influence of a single factor, which is the level of the water salinity, on the microbiota composition. The trophic level also has an effect on the microbiota; the microbiota of herbivorous fishes (including common carp) typically resemble the microbiota of mammals [16]. Pyrosequencing of 16S rRNA revealed that the gastrointestinal microbiota of common carp contains *Fusobacteria* (46%), *Bacteroidetes* (21%), *Planctomycetes* (12%), *Gammaproteobacteria* (7%), as well as *Clostridia* (3%), *Verrucomicrobiae* (1%), and *Bacilli* (1%) [17]. 

The gastrointestinal microbiota can be modulated by prebiotics supplemented in feed. The most commonly used prebiotics in aquaculture include inulin, beta-glucan, fructooligosaccharides (FOS), mannanoligosaccharides (MOS), galactooligosaccharides (GOS), xylooligosaccharides (XOS), arabinoxyligosaccharides (AXOS), and isomaltooligosaccharides (IMO) [18]. The use of a prebiotic or synbiotic (prebiotic + probiotic) in the fish’s diet improves growth parameters [19,20], stimulates digestive enzymes [21], increases resistance to bacterial and viral diseases [22], improves hematological parameters [23], modulates composition of the intestinal microbiota, enhances intestinal microvilli and absorption surface [19,24,25,26], and also affects the level of the immune-related gene expression [27]. 

Modulating the innate immune responses in fish by feed additives can support health and prevent diseases [19]. Various feed additives with immunostimulatory effects have been studied, such as herbs, prebiotics, probiotics, and synbiotics [20,28,29,30]. The mucus layers of the fish skin are rich in ingredients associated with the innate immune system, such as immunoglobulins, complement proteins, c-reactive proteins, lysozymes, proteases, and antimicrobial peptides [31]. The aim of the study was to analyze the effects of GOS prebiotic applied as a feed additive on the mRNA expression of the genes associated with the innate immune responses in the skin mucosa of the common carp (*Cyprinus carpio*). 

## 2. Materials and Methods

### 2.1. Fish, Feeds, and Experimental Design

The experiment was conducted at the Experimental Station of Feed Production Technology and Aquaculture (affiliated with Poznań University of Life Sciences) in Muchocin (Poland). Three hundred one-year-old fish of the common carp (*Cyprinus carpio*), with a mean bodyweight of 180 g (±5 g), were placed in 12 concrete tanks (60 m^3^). The distribution of the fish was 25 individuals per tank. The tanks were individually supplied with water from the Struga Dormowska river, in an open system with a mechanical prefiltration chamber. Construction of the tanks allowed the maintenance of the maximum water level with constant water flow. Each tank was equipped with an automatic belt feeder allowing permanent access to the feed for 12 h a day.

The daily diet ration was calculated based on the carp feeding key developed by Miyatake [32]. Water temperature and the current fish weight was accounted for. The dietary formulation and proximate composition of the feeds are shown in Table 1. The feeds were processed using a single-screw warm extruder (Metalchem S-60, Gliwice, Poland). The extrusion conditions were as follows: cylinder temperature under stress of increasing pressure 90 °C, 100 °C in the high-pressure zone, 110 °C in the head, with a screw diameter of 6 mm and a speed of 52 rpm. The fish were divided into two groups: control group (CON), which received a diet without supplements, and experimental group (GOS), fed a diet supplemented with 2% GOS (Bi_2_tos^®^, Clasado Biosciences Ltd., Jersey, UK). The feeding trial lasted 50 days, from 30.04. to 19.06.2018. Every ten days, individual weights of all fish in each tank were measured, and the feed ration and rearing indices were calculated.

### 2.2. Tissue Collection and RNA Isolation 

Samples of the skin mucosa were collected from randomly selected individuals (n = 8) with an average body weight of 500 g (±10 g). The mucus was gently scraped from the skin using a sterile glass slide and stabilized in 3 ml of RNA*later* (Invitrogen, Waltham, MA, USA). The samples were stored at −80 °C until total RNA isolation. Prior to total RNA isolation, the samples of skin mucosa were homogenized with the TissueRuptor homogenizer (Qiagen GmbH, Hilden, Germany) in TRIzol® LS Reagent (Ambion/Thermo Fisher Scientific, Waltham, MA, USA). The lysate was processed using a EURx Universal RNA Purification Kit (EURx, Gdansk, Poland). The RNA quality and quantity were determined by gel electrophoresis using 2% agarose gel (to verify the integrity of 18S and 28S rRNA) and NanoDrop 2000 (to measure the absorbance at 260/280 nm) (Scientific Nanodrop Products, Wilmington, DE, USA). RNA was frozen at -80 °C prior to downstream analyses.

### 2.3. Reverse Transcription–Quantitative PCR (RT-qPCR)

Reverse transcription (RT) was performed using a Maxima First Strand cDNA Synthesis Kit for RT-qPCR (Thermo Scientific/Fermentas, Vilnius, Lithuania), following the manufacturer’s recommendations. Obtained cDNA was diluted to 70 ng/μL and stored at −20 °C. RT-qPCR reactions were conducted with a total volume of 10 μL. The reaction mixture contained 1x Maxima SYBR Green qPCR Master Mix (Thermo Scientific/Fermentas, Vilnius, Lithuania), 1 μM of each primer (Sigma–Aldrich, Germany), and 2 μl of diluted cDNA. Thermal cycling was performed in a LightCycler II 480 (Roche Diagnostics, Basel, Switzerland). The qPCR amplification comprised an initial denaturation step for 15 min at 95 °C, followed by 40 cycles of denaturation (10 s at 95 °C), annealing (15 s at 58 °C), and extension (30 s at 72 °C). Fluorescence was measured at the end of each extension step. The thermal program was completed by the melting curve, which was generated by increasing the temperature in small increments up to 98 °C and measuring the fluorescence of the melting amplicon. Each RT-qPCR reaction was conducted in triplicates (reference genes) or duplicates (target genes). Oligonucleotide primers were synthesized based on sequences from literature or in-house designed. The selection of reference and target genes and primer details are described in the Section 2.4. “Gene selection”.

### 2.4. Gene Selection

#### 2.4.1. Reference Genes

Reference genes for the relative gene expression analysis were selected based on a two-step selection process. First, the related literature was studied to pinpoint the relevant panel of reference genes for carp [24,33,34,35]. Second, the RT-qPCR analysis was performed on a full set of cDNA samples to determine the reference genes’ quality and stability in the samples from skin mucosa. The RT-qPCR for reference genes was performed based on the methodology described in Section 2.3. “Reverse Transcription Quantitative PCR (RT-qPCR)”. Table 2 presents the list of the reference genes and the respective oligonucleotide primers. Ct values from CON and GOS groups were analyzed using RefFinder [36]. RefFinder integrates different algorithms that are commonly used in reference genes analysis, including BestKeeper [37], NormFinder [38], geNorm [39], and the comparative delta-Ct method [40]. Analysis of the panel of reference genes allowed for selecting the best combination of the reference genes for relative expression of the target genes.

#### 2.4.2. Target Genes

The selection of the target genes was based on the literature on the immune responses generated in SALT [5,41,42,43,44]. First, a list of immunological processes that occur in SALT to protect fish from external factors was determined. These processes include defense against Gram-positive bacteria, Gram-negative bacteria, viruses, fungi, and yeast; hydrolyzing peptide and glycosidic bonds of the cell walls; promoting phagocytosis; activating complement pathways; turning off opsonization; and stress response. Next, proteins involved in these processes were pinpointed, and the underlying genes were considered target genes for this study. In effect, a comprehensive panel of the genes expressed in the fish skin mucosa was selected. A DNA sequence of the respective target genes was derived from a gene-related section of the NCBI database (https://www.ncbi.nlm.nih.gov/gene). The RT-qPCR primers were designed using Primer-BLAST [45], which is a primer designing tool implemented in the NCBI database (https://www.ncbi.nlm.nih.gov/tools/primer-blast/index.cgi). The RT-qPCR primers were designed based on the following criteria: amplicon size between 70 and 200 bp, span of an exon–exon junction (does not apply to *CRP* gene due to the presence of only one exon), optimal melting temperatures around 60 °C, the 3’ end of primers contains a C or G residue (if possible), CG content around 40%–60%, and exclusion of primer–dimer formation. The list of the analyzed genes, including their biological function and primer sequences, is presented in Table 3.

### 2.5. Relative Quantification of Gene Expression and Statistical Analysis

Normalization of the expression levels (Ct values) of the target genes was performed with two selected reference genes (*ACTB* and *40s s11*). The geometric mean of Ct values between reference genes was used to calculate ∆Ct, according to the formula: ∆Ct = Ct target – Ct reference. Relative gene expression was calculated with the ∆∆Ct algorithm, in which CON was considered a calibrator. The fold change in the target genes in GOS was calculated as 2^–∆∆*C*t^ [48]. To compare the data between GOS and CON, a Student’s *t*-test was performed using the SAS Enterprise Guide 9.4 program (SAS Institute, Cary, NC, USA). The difference between GOS and CON was considered significant when *p* < 0.05. Figures were prepared using GraphPad Prism 7 (GraphPad, La Jolla, CA, USA).

## 3. Results

### 3.1. Reference Genes

**Figure 1:**
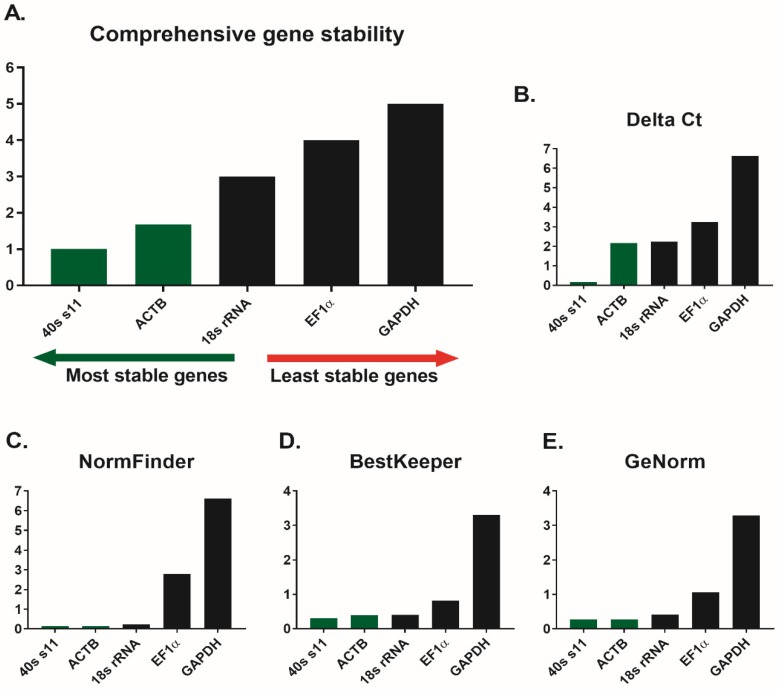
Analyses of the candidate reference genes for gene expression study in skin mucosa of common carp (*Cyprinus carpio*) using different algorithms: (**A**) comprehensive gene stability, (**B**) Delta Ct, (**C**) Normfinder, (**D**) BestKeeper, and (**E**) GeNorm. Candidate reference genes: Beta-actin (ACTB), Elongation factor 1-alpha (EF-1α), Glyceraldehyde-3-phosphate dehydrogenase-like (GAPDH), 18S ribosomal RNA (18s rRNA), and 40s ribosomal protein s11 (40s s11). Dataset was generated for GOS-supplemented and control animals (n = 8) using RT-qPCR. qPCR reactions were performed in triplicates. RefFinder was used to calculate the gene stability values. 40s s11 and ACTB (labeled green) were selected as the most stable pair of reference genes for skin mucosa study in carp. Figures were prepared using GraphPad Prism 7 (GraphPad, La Jolla, CA, USA).

The results of the reference genes used for relative gene expression analysis in skin mucosa of common carp are presented in Figure 1. All computational methods (the comparative delta-Ct method, BestKeeper, NormFinder, and GeNorm) showed that the two genes that are most stable in the carp skin mucosa are: *40s s11* and *ACTB*. In addition, GeNorm indicated that the best set of the candidate genes for normalization of the experiment would be the geometric mean of the *ACTB*/*40s s11* genes. In the following calculations of the relative immune-related gene expression analysis, the geometric mean of those two reference genes was used.

### 3.2. Immune-Related Gene Expression

**Figure 2:**
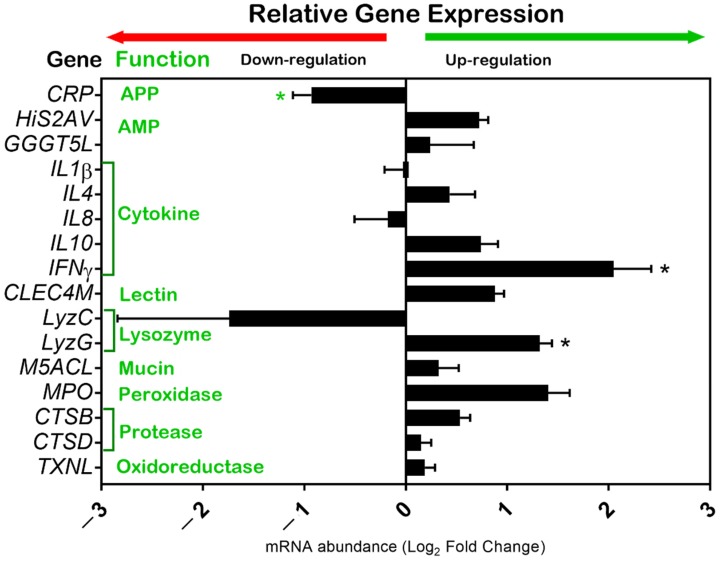
Immune-related gene expression signatures identified in the skin mucosa of common carp (*Cyprinus carpio*) supplemented with GOS. The *Y*-axis shows a list of genes (black) and enzymatic function of encoded proteins (green). Horizontal bars on the X-axis indicate the relative mRNA abundance of the genes of GOS-supplemented animals (n = 8). Gene expression analysis was carried out with RT-qPCR. qPCR reactions were performed in triplicates. The geometric mean of the 40s s11 and ACTB reference genes was used to calculate dCt values. The relative gene expression was calculated with the ddCt formula (FC = 2^–ΔΔ*C*t^). FC values were transformed and presented as Log_2_FC. The standard error of the means (SEM) shows the distribution of the Ct values. Normalized data (dCt values) of control and treated groups were compared with the Student’s *t*-test. Significant differences (*p* < 0.05) were labeled with an asterisk (*). Figures were prepared using GraphPad Prism 7 (GraphPad, La Jolla, CA, USA). Abbreviations used in the figure: APP—acute-phase proteins; AMP—antimicrobial proteins.

The relative gene expression analysis for immune-related genes in the carp skin mucosa is shown in Figure 2. The majority of the genes were upregulated in GOS vs. CON. Only CRP and LyzC were downregulated. Statistically significant differences at the mRNA level were demonstrated for INFγ and LyzG genes, which were upregulated in GOS (*p* ≤ 0.05). The CRP gene was significantly downregulated in GOS (*p* ≤ 0.01). Furthermore, a suggestive statistical trend was found for the MPO gene, which was upregulated in GOS compared to CON (0.05 < *p* <0.1). 

## 4. Discussion

Expression of the immune-related genes gives insight into the mechanisms of the innate immune responses. In aquaculture, replacing antibiotics with prebiotics, probiotics, or synbiotics, increases immunological competence and resistance to diseases in an environmentally friendly way [49]. The immunomodulatory role of prebiotics results from direct interactions with the innate immune system and/or indirectly, by selectively stimulating the growth of commensals inhabiting the host’s mucosa [50,51]. This way, prebiotics can stimulate intestinal epithelial cells to release cytokines that modulate the spectrum of the mucosal immune system, including dendritic cells, T cells, and B cells, which, in turn, trigger the transcription of immune-related genes (e.g., tumor necrosis factor α, or lysozyme). This process leads to an increase in innate immune responses [52,53].

### 4.1. mRNA Expression Stability of the Reference Genes

One of the most important factors that can skew the results of the relative gene expression at the mRNA level is the selection of the most stable reference genes for a given tissue. To our knowledge, the information regarding the most suitable genes for normalizing the RT-qPCR data in the skin mucosa of common carp was lacking. In fish, the most common reference genes are *ACTB* [54], *B2M*, *18s rRNA*, *EF1α*, and *GAPDH* [55,56,57,58]. This diversity may be due to the species characteristics, individual tissues, age, and type of experiment. Thus, it is necessary to test and compare different housekeeping genes in all experimental conditions [59]. In this study, five candidate genes were evaluated for the normalization of RT-qPCR in the skin mucosa of the common carp. The highest expression stability in skin mucosa was found for *ACTB* and *40s s11* genes. Gene *ACTB* encodes actin. All actins are highly conserved and involved in cell motility, structure, integrity, and intercellular signaling. ACTB is a protein found in most vertebrate cells as components of the cytoskeleton (https://www.genecards.org/). The protein encoded by the *40s s11* gene is a member of the S17P family of ribosomal proteins. This family is a component of the small ribosome subunit (40S). The main function of this gene is binding RNA and rRNA. Protein *40s s11* is also a structural component of the ribosome (https://www.genecards.org/). In our experiment, the mRNA expression of *ACTB* and *40s s11* genes was not affected by the experimental factor (GOS). For this reason, they are good candidates for internal control genes in RT-qPCR experiments. 

### 4.2. Immune-Related Gene Expression in Skin Mucosa

The obtained results of the immune-related gene expression in response to GOS supplementation showed a significant increase in the genes associated with antiviral (*IFNγ*) and antimicrobial (*LyzG*) immune responses. On the other side, the *CRP* gene representing the acute phase response, was decreased in the GOS-supplemented group. IFNγ is an antiviral and immunoregulatory cytokine that is necessary for cellular defense. It is produced by T cells and natural killer cells as a dimerized soluble glycoprotein [60]. Lysozymes are an important element of innate immunity. They are able to catalyze hydrolysis of the bacterial glycosidic bonds. Several types of lysozymes have been described, such as lysozyme C (chicken), lysozyme G (goose), phage, bacterial and plant lysozymes [61]. In fish, lysozyme is found in mucus, serum, and ova [62] and is found in two forms, C and G. Fish lysozymes are thought to have a much more bactericidal effect than those produced by the higher vertebrates [61]. CRP belongs to the family of soluble proteins that are involved in the acute phase reaction (APR) to injury, damage, or infection. CRP is able to bind to phosphorylcholine, pneumococcal C-polysaccharide, and phospholipids, as well as to autogenic compounds, such as apoptotic nuclear components and other intracellular components released after cell death [63]. It also binds harmful molecules, such as mercury, increases phagocytic clearance, and triggers complement activation via the classical pathway [64].

### 4.3. Effects of GOS in Fish

Several papers have reported improvement in the immunological properties of the fish skin mucosa after application of the GOS prebiotic. They refer to the increase in the activity of immunological factors at the protein level in the skin mucosa. Hoseinifar et al. [65] compared the effects of three prebiotics (FOS, GOS, and inulin) in common carp. A significant increase in dermal lysozyme activity in the experimental group fed GOS was demonstrated, which is in line with the present study. Hoseinifar et al. [23] demonstrated similar effects in rainbow trout (*Oncorhynchus mykiss*) fed diets supplemented with 2% GOS. The supplementation of GOS (1%–2%) in the nutrition of the white Caspian fish (*Rutilus frisii kutum*) for eight weeks also increased the lysozyme activity and total immunoglobulin level [66]. The introduction of XOS in diets fed to the white Caspian fish also increased bactericidal activity in the skin mucus [67]. In goldfish (*Carassius auratus auratus*), 1% and 2% GOS significantly improved the immune parameters of the skin mucus (lysozyme and total protein) compared to the control and to the reduced proportion of GOS in the mix (0.5%) [68]. These studies suggest that dietary prebiotics (including GOS) indirectly support mucosal immunity. However, further research is needed on total IgA initiation by prebiotics in fish to fully understand the effect of immunomodulation [69].

The effects of GOS supplemented to the fish also improved intestinal function and muscle structure in common carp (*Cyprinus carpio*). The feeding trial reported in this paper lead to the discovery that GOS provided in the diet improved the histological picture of the intestines, including the height and thickness of intestinal villi. Such morphological changes in the fish guts increased the absorptive surface of the small intestine. The elevated ratio of villi height to intestinal crypt depth suggests improved maturity of the intestinal mucosa of the GOS-supplemented carp diet (Ziółkowska et al., submitted). Regarding the muscle structure, the addition of 2% GOS increased the diameter and density of the white muscle fibers responsible for the marbling of the fish meat. Along with that, the percentage of muscle fiber atrophy decreased (Ziółkowska, personal communication). In conclusion, GOS supplementation in carp improved intestinal and muscular morphology.

### 4.4. Immunomodulatory Role of GOS

GOS used in this study was produced from lactose by galactosyltransferases from *Bifidobacterium bifidum* NCIMB 41,171 isolated from a human stool sample [70]. The immunomodulatory effects of this particular compound have been well-established in human and poultry. The introduction of GOS to human diets increased fecal bifidobacteria abundance while reducing less desirable strains [71,72,73]. Dietary GOS significantly increased fagocytosis, stimulated NK activity, increased levels of anti-inflammatory cytokine (IL-10) and decreased levels of pro-inflammatory cytokines (IL-6, IL-1, and TNF-α) in elderly people [72]. Supplementation of GOS in diets of overweight adults led to an increased abundance of colonic *Bifidobactera*, as well as increased production of fecal secretory IgA [71]. GOS has also been reported to alleviate the syndromes of irritable bowel syndrome [74], prevented the symptoms of traveler’s diarrhea [75], and exerted positive effects on GI symptoms, including bloating, abdominal pain, and flatulence [76].

The immunomodulatory effects of GOS used for early stimulation of the chicken microbiota through *in ovo* technology have been widely documented [77,78,79]. Transcriptomic analysis revealed that GOS delivered *in ovo* modulated genes associated with lymphocyte proliferation, activation and differentiation, as well as cytokine production in the caecal tonsils of broiler chickens [78]. It was also found that GOS delivered *in ovo* increased expression of the genes related to mucosal immune response, intestinal barrier function, and nutrient sensing in the chicken gastrointestinal tract [77]. Particularly beneficial effects of GOS delivered *in ovo* were determined during heat stress in broiler chickens. Even a short-term increase in ambient temperatures resulted in elevated expression of the genes associated with immune response and stress response [80]. These effects were alleviated by GOS delivered *in ovo*, most likely due to maintaining intestinal eubiosis [80]. Furthermore, GOS delivered *in ovo* mitigated harmful effects of chronic heat stress on the performance and welfare of broiler chickens [81], as well as meat composition and quality [82]. 

## 5. Conclusions

Supplementation of the standard diets with GOS modulated innate immune responses of common carp. In this study, we found that dietary GOS exerted immunomodulatory effects on skin mucosa, which was manifested by mRNA expression of the genes involved in cytokine, lysozyme, and acute-phase protein production. In conclusion, GOS activated immunomodulatory pathways leading to gene expression modulation in SALT of common carp.

## Figures and Tables

**Table 1:** Dietary formulation and proximate composition of the feed.

Ingredient	Composition (%)	
	CON ^11^	GOS ^12^
Wheat meal	32.8	30.8
Fish meal ^1^	12.3	12.3
Blood meal ^2^	10.0	10.0
DDGS ^3^	11.0	11.0
Soybean meal ^4^	15.0	15.0
Rapeseed meal ^5^	10.0	10.0
Fish oil ^6^	4.6	4.6
Soybean lecithin ^7^	1.0	1.0
Vitamin-mineral premix ^8^	1.5	1.5
Vitamin premix ^9^	0.1	0.1
Choline chloride	0.2	0.2
Fodder chalk	1.5	1.5
Prebiotic ^10^	0	2
**Proximate composition (% dry matter)**		
Crude protein	35.06	
Essential amino acids (g 100 g ^−1^ of crude protein) Arginine	4.53	
Histidine	2.80	
Lysine	3.50	
Tryptophan	1.04	
Phenylalanine + Tyrosine	4.96	
Methionine + Cysteine	1.75	
Threonine	3.13	
Leucine	6.72	
Isoleucine	3.90	
Valine	4.97	
Total lipid	9.08	
Crude fiber	3.93	
Total phosphorus	0.83	
Calcium	1.36	
Ash	7.17	
Gross energy (MJ·kg ^−1^)	18.51	

^1^ Danish fishmeal, Type F, 72% total protein, 12% fat, FF Skagen, Denmark. ^2^ AP 301 P, 92% total protein, APC (GB) Ltd, Ings Road, Doncaster, UK. ^3^ Dried Distillers Grains with Solubles, stillage >45% total protein, <6% ash. ^4^ Toasted, 46%–47% total protein. ^5^ 33% total protein, 2% fat. ^6^ Agro-fish, Kartoszyno, Poland. ^7^ BergaPure, deoiled lecithin, 97% pure lecithin, Berg+Schmidt GmbH & Co. KG, Hamburg, Germany. ^8^ Polfamix W, BASF Polska Ltd. Kutno, Poland – 1 kg contains: vitamin A 1,000,000 IU, vitamin D_3_ 200,000 IU, vitamin E 1.5 g, vitamin K 0.2 g, vitamin B_1_ 0.05 g, vitamin B_2_ 0.4 g, vitamin B_12_ 0.001 g, nicotinic acid 2.5 g, D-calcium pantothenate 1.0 g, choline chloride 7.5 g, folic acid 0.1 g, methionine 150.0 g, lysine 150.0 g, Fe 2.5 g, Mn 6.5 g, Cu 0.8 g, Co 0.04 g, Zn 4.0 g, J 0.008 g, carrier up to 1000.0 g. ^9^ Vitazol AD_3_E, BIOWET Drwalew, Poland – 1 kg contains: vitamin A 50,000 IU, vitamin D_3_ 5000 IU, vitamin E 30.0 mg, vitamin C 100.0 mg. ^10^ Bitos® trans-galactooligosaccharide (GOS), Clasado Ltd. ^11^ Control group without GOS supplementation. ^12^ Group supplemented with galactooligosaccharides.

**Table 2:** Reference genes for reverse transcription–quantitative PCR (RT-qPCR) in common carp.

Name	Gene	NCBI Gene ID	Primer Sequences (5’→3’)	Ref
Beta-actin	*ACTB*	109073280	F:ATCCGTAAAGACCTGTATGCCA R:GGGGAGCAATGATCTTGATCTTCA	[24]
Elongation factor 1-alpha	*EF-1α*	109111735	F:TGGAGATGCTGCCATTGT R:TGCAGACTTCGTGACCTT	[34]
Glyceraldehyde-3-phosphate dehydrogenase-like	*GAPDH*	109106399	F:ATCTGACGGTCCGTCT R:CCAGCACCGGCATCAAA	[34]
18S ribosomal RNA	*18s rRNA*	FJ710826.1	F:GAGTATGGTTGCAAAGCTGAAAC R:AATCTGTCAATCCTTTCCGTGTCC	[35]
40S ribosomal protein S11	*40s s11*	109061205	F:CCGTGGGTGACATCGTTACA R:TCAGGACATTGAACCTCACTGTCT	[33]

**Table 3:** Immune-related genes and primer sequences for RT-qPCR analysis of the skin mucosa in common carp.

Name	Gene	Gene ID	Function ^1^	Primer Sequences (5’→3’)	Ref. ^2^
Acute-phase protein					
C-reactive protein	*CRP*	109083752	Host defense: it promotes agglutination, bacterial capsular swelling, phagocytosis, and complement fixation through its calcium-dependent binding to phosphorylcholine.	F:AGCTTTGGAAAATTCGGTTCACC R:ACTCACCCTCGTGTCACTGC	This study
Antimicrobial peptides (AMP)					
Histone H2A.V-like	*His2Av*	109068402	Main role in transcription regulation, DNA repair, DNA replication, and chromosomal stability	F:CTGGTGGAGGTGTGATTCCT R:AGCGGGAACTACACGGTCTT	This study
Protein-glutamine gamma-glutamyltransferase 5-like	*GGGT5L*	109112827	Key role in the gamma-glutamyl cycle and maintains normal redox status	F:AGCTGCATATCATGGACGAGTT R:CTCCGCAGAACCAGAGTGCT	This study
Cytokines					
Interleukin 1 beta-like	*IL1β*	109097442	Mediator of the inflammatory response, and is involved in a variety of cellular activities, including cell proliferation, differentiation, and apoptosis	F:AAGGAGGCCAGTGGCTCTGT R:CCTGAAGAAGAGGAGGCTGTCA	[46]
Interleukin 4	*IL4*	109064937	Participates in at least several B-cell activation processes as well as other cell types. It is a costimulator of DNA-synthesis. It induces the expression of class II MHC molecules on resting B-cells	F:TTTCTGGGCTGTCTGGTGCCAA R:TTTCTTGTCAGTACGGAAATGCTCA	[47]
Interleukin 8-like	*IL8*	109085034	Chemotactic factor that attracts neutrophils, basophils, and T-cells, but not monocytes. It is also involved in neutrophil activation. It is released from several cell types in response to an inflammatory stimulus	F:GATGCAAATGCCCTCAAATACA R:GGCTCTTGACGTTCCTTTTG	[43]
Interleukin 10-like	*IL10*	109076801	Major immune-regulatory cytokine that acts on many cells of the immune system where it has profound anti-inflammatory functions, limiting excessive tissue disruption caused by inflammation	F:CGCCAGCATAAAGAACTCGT R:TGCCAAATACTGCTCGATGT	[46]
Interferon gamma	*IFNγ*	109053615	Produced by lymphocytes activated by specific antigens or mitogens	F:TGAGCTTAAAGAATGTGTGGCCCAA R:ACTCCATATGTGACGGCTTTTGGT	[47]
Lectins					
C-type lectin 4	*CLEC4M*	109066444	Binds carbohydrates mannose and fucose	F:TCAACTGGTCAGAGGCACGA R:GAAAGGCCCACTCTTCATCGTC	This study
Lyzosymes					
Lyzosyme C	*LyzC*	109090952	Protection against pathogens	F:ATGAAGGTGACTATTGCTGTCTTG R:AGTAGGCCGTGCACACATAGTT	This study
Lyzosyme G	*LyzG*	109087581	Protection against pathogens	F:GGCCTTCAGACGATACTTACCA R:TGGAAGCCTCGACACCCTTT	This study
Mucins					
Mucin-5AC-like	*M5ACL*^3^ (LOC109110796)	109110796	Forming protective mucous barriers on epithelial surfaces	F:CGATCAGTGCTATGTCCTGTCA R:ACAGTTGGGCTCACGTTTGT	This study
Peroxidases					
Myeloperoxidase-like	*MPO*	109052003	Produces hypochlorous acid from hydrogen peroxide and chloride anion during the neutrophil’s respiratory burst, oxidizes tyrosine to the tyrosyl radical using hydrogen peroxide as an oxidizing agent	F:CAACCTGGTCCACAAGGTGTAGC R:GGCAGACTGTTGTCCTGTGG	This study
Proteases					
Cathepsin B	*CTSB*	109064698	Bacteriolytic activity against fish pathogen	F:CACTGACTGGGGTGATAATGGATA R:GGTGCTCATTTCAGCCCTCCT	This study
Cathepsin D	*CTSD*	109105685	Regulates production of parasin I	F:CGACGGCTCGCCAAAATGAG R:AGAGGAATCCGTACAATTGCGT	This study
Oxidoreductase					
Thioredoxin-like	*TXNL*^3^ (LOC109108046)	109108046	Cell redox homeostasis	F:GCGGGCTGCTGCTTTGACTG R:GTCGAAGGCAGGCTTATCCTCA	This study
Reference genes					
Beta-actin	*ACTB*	109073280	Actins are highly conserved proteins that are involved in cell motility, structure, integrity, and intercellular signaling	F:ATCCGTAAAGACCTGTATGCCA R:GGGGAGCAATGATCTTGATCTTCA	[24]
40S ribosomal protein S11	*40s s11*	109061205	Relation with viral mRNA translation and activation of the mRNA pathways upon binding of the cap-binding complex and eIFs, and subsequent binding to 43S	F:CCGTGGGTGACATCGTTACA R:TCAGGACATTGAACCTCACTGTCT	[33]

^1^ gene function derive from GeneCards (http://www.genecards.org); ^2^ primers marked as “this study” were designed using Primer-BLAST [45]; ^3^ name given for this experiment.

## References

[1] Wang S., Wang Y., Ma J., Ding Y., Zhang S. (2011). Phosvitin plays a critical role in the immunity of zebrafish embryos via acting as a pattern recognition receptor and an antimicrobial effector. J. Biol. Chem..

[2] Hawkes J.W. (1974). The structure of fish skin—I. General organization. Cell Tissue Res..

[3] Fast M., Sims D., Burka J., Mustafa A., Ross N. (2002). Skin morphology and humoral non-specific defence parameters of mucus and plasma in rainbow trout, coho and Atlantic salmon. Comp. Biochem. Physiol. A. Mol. Integr. Physiol..

[4] Arasu A., Kumaresan V., Sathyamoorthi A., Palanisamy R., Prabha N., Bhatt P., Roy A., Thirumalai M.K., Gnanam A.J., Pasupuleti M. (2013). Fish lily type lectin-1 contains β-prism architecture: Immunological characterization. Mol. Immunol..

[5] Swain P., Dash S., Sahoo P., Routray P., Sahoo S., Gupta S., Meher P., Sarangi N. (2007). Non-specific immune parameters of brood Indian major carp Labeo rohita and their seasonal variations. Fish Shellfish Immunol..

[6] Ángeles Esteban M. (2012). An Overview of the Immunological Defenses in Fish Skin. ISRN Immunol..

[7] Lazado C.C., Caipang C.M.A. (2014). Bacterial viability differentially influences the immunomodulatory capabilities of potential host-derived probiotics in the intestinal epithelial cells of Atlantic cod *Gadus morhua*. J. Appl. Microbiol..

[8] Streilein J.W. (1983). Skin-associated lymphoid tissues (SALT): Origins and functions. J. Invest. Dermatol..

[9] Xu Z., Parra D., Gomez D., Salinas I., Zhang Y.-A., von Gersdorff Jorgensen L., Heinecke R.D., Buchmann K., LaPatra S., Sunyer J.O. (2013). Teleost skin, an ancient mucosal surface that elicits gut-like immune responses. Proc. Natl. Acad. Sci. USA.

[10] Austin B. (2006). The bacterial microflora of fish, revised. Sci. World J..

[11] Musharrafieh R., Tacchi L., Trujeque J., LaPatra S., Salinas I. (2014). Staphylococcus warneri, a resident skin commensal of rainbow trout (Oncorhynchus mykiss) with pathobiont characteristics. Vet. Microbiol..

[12] Boutin S., Bernatchez L., Audet C., Derôme N. (2012). Antagonistic effect of indigenous skin bacteria of brook charr (Salvelinus fontinalis) against Flavobacterium columnare and F. psychrophilum. Vet. Microbiol..

[13] Carbajal-González M., Fregeneda-Grandes J., Suárez-Ramos S., Rodríguez Cadenas F., Aller-Gancedo J. (2011). Bacterial skin flora variation and in vitro inhibitory activity against Saprolegnia parasitica in brown and rainbow trout. Dis. Aquat. Organ..

[14] Horsley R.W. (1973). The bacterial flora of the Atlantic salmon (Salmo salar L.) in relation to its environment. J. Appl. Bacteriol..

[15] Cahill M.M. (1990). Bacterial flora of fishes: A review. Microb. Ecol..

[16] Sullam K.E., Essinger S.D., Lozupone C.A., O’Connor M.P., Rosen G.L., Knight R., Kilham S.S., Russell J.A. (2012). Environmental and ecological factors that shape the gut bacterial communities of fish: A meta-analysis. Mol. Ecol..

[17] van Kessel M.A.H.J., Dutilh B.E., Neveling K., Kwint M.P., Veltman J.A., Flik G., Jetten M.S.M., Klaren P.H.M., Op den Camp H.J.M. (2011). Pyrosequencing of 16s rRNA gene amplicons to study the microbiota in the gastrointestinal tract of carp (Cyprinus carpio L.). AMB Express.

[18] Kazuñ B., Kazuñ K., Siwicki A.K. (2016). Probiotyki, prebiotyki i synbiotyki w ochronie zdrowia ryb. Komunikaty Rybackie.

[19] Hoseinifar S.H., Khalili M., Khoshbavar Rostami H., Esteban M.Á. (2013). Dietary galactooligosaccharide affects intestinal microbiota, stress resistance, and performance of Caspian roach (Rutilus rutilus) fry. Fish Shellfish Immunol..

[20] Do Huu H., Jones C.M. (2014). Effects of dietary mannan oligosaccharide supplementation on juvenile spiny lobster Panulirus homarus (Palinuridae). Aquaculture.

[21] Guerreiro I., Enes P., Rodiles A., Merrifield D., Oliva-Teles A. (2016). Effects of rearing temperature and dietary short-chain fructooligosaccharides supplementation on allochthonous gut microbiota, digestive enzymes activities and intestine health of turbot ( *Scophthalmus maximus* L.) juveniles. Aquac. Nutr..

[22] Luna-González A., Almaraz-Salas J.C., Fierro-Coronado J.A., Flores-Miranda M. del C., González-Ocampo H.A., Peraza-Gómez V. (2012). The prebiotic inulin increases the phenoloxidase activity and reduces the prevalence of WSSV in whiteleg shrimp (Litopenaeus vannamei) cultured under laboratory conditions. Aquaculture.

[23] Hoseinifar S.H., Mirvaghefi A., Amoozegar M.A., Merrifield D.L., Ringø E. (2015). In vitro selection of a synbiotic and in vivo evaluation on intestinal microbiota, performance and physiological response of rainbow trout (Oncorhynchus mykiss) fingerlings. Aquac. Nutr..

[24] Liu W., Yang Y., Zhang J., Gatlin D.M., Ringø E., Zhou Z. (2014). Effects of dietary microencapsulated sodium butyrate on growth, intestinal mucosal morphology, immune response and adhesive bacteria in juvenile common carp (Cyprinus carpio) pre-fed with or without oxidised oil. Br. J. Nutr..

[25] Torrecillas S., Makol A., Benítez-Santana T., Caballero M.J., Montero D., Sweetman J., Izquierdo M. (2011). Reduced gut bacterial translocation in European sea bass (Dicentrarchus labrax) fed mannan oligosaccharides (MOS). Fish Shellfish Immunol..

[26] Grześkowiak Ł., Collado M.C., Vesterlund S., Mazurkiewicz J., Salminen S. (2011). Adhesion abilities of commensal fish bacteria by use of mucus model system: Quantitative analysis. Aquaculture.

[27] Rungrassamee W., Kingcha Y., Srimarut Y., Maibunkaew S., Karoonuthaisiri N., Visessanguan W. (2014). Mannooligosaccharides from copra meal improves survival of the Pacific white shrimp (Litopenaeus vannamei) after exposure to Vibrio harveyi. Aquaculture.

[28] Hoseinifar S.H., Ringø E., Shenavar Masouleh A., Esteban M.Á. (2016). Probiotic, prebiotic and synbiotic supplements in sturgeon aquaculture: A review. Rev. Aquac..

[29] Khalil S.R., Reda R.M., Awad A. (2017). Efficacy of Spirulina platensis diet supplements on disease resistance and immune-related gene expression in Cyprinus carpio L. exposed to herbicide atrazine. Fish Shellfish Immunol..

[30] Ringø E., Song S.K. (2016). Application of dietary supplements (synbiotics and probiotics in combination with plant products and β-glucans) in aquaculture. Aquac. Nutr..

[31] Subramanian S., MacKinnon S.L., Ross N.W. (2007). A comparative study on innate immune parameters in the epidermal mucus of various fish species. Comp. Biochem. Physiol. Part B Biochem. Mol. Biol..

[32] Miyatake H. (1997). Carp. Yoshoku.

[33] Metz J.R., Huising M.O., Leon K., Verburg-van Kemenade B.M.L., Flik G. (2006). Central and peripheral interleukin-1 and interleukin-1 receptor I expression and their role in the acute stress response of common carp, Cyprinus carpio L.. J. Endocrinol..

[34] Shrivastava J., Rašković B., Blust R., De Boeck G. (2018). Exercise improves growth, alters physiological performance and gene expression in common carp (Cyprinus carpio). Comp. Biochem. Physiol. Part A Mol. Integr. Physiol..

[35] Zhang W., Jia Y., Ji X., Zhang R., Liang T., Du Q., Chang Z. (2016). Optimal reference genes in different tissues, gender, and gonad of Yellow River carp (Cyprinus carpio var) at various developmental periods. Pak. J. Zool..

[36] Xie F., Xiao P., Chen D., Xu L., Zhang B. (2012). miRDeepFinder: A miRNA analysis tool for deep sequencing of plant small RNAs. Plant Mol. Biol..

[37] Pfaffl M.W., Tichopad A., Prgomet C., Neuvians T.P. (2004). Determination of stable housekeeping genes, differentially regulated target genes and sample integrity: BestKeeper – Excel-based tool using pair-wise correlations. Biotechnol. Lett..

[38] Andersen C.L., Jensen J.L., Ørntoft T.F. (2004). Normalization of real-time quantitative reverse transcription-PCR data: A model-based variance estimation approach to identify genes suited for normalization, applied to bladder and colon cancer data sets. Cancer Res..

[39] Vandesompele J., De Preter K., Pattyn F., Poppe B., Van Roy N., De Paepe A., Speleman F. (2002). Accurate normalization of real-time quantitative RT-PCR data by geometric averaging of multiple internal control genes. Genome Biol..

[40] Silver N., Best S., Jiang J., Thein S. (2006). Selection of housekeeping genes for gene expression studies in human reticulocytes using real-time PCR. BMC Mol. Biol..

[41] Dash S., Das S.K., Samal J., Thatoi H.N. (2018). Epidermal mucus, a major determinant in fish health: A review. Iran. J. Vet. Res..

[42] Dawar F.U., Tu J., Xiong Y., Lan J., Dong X.X., Liu X., Khattak M.N.K., Mei J., Lin L. (2016). Chemotactic Activity of Cyclophilin A in the Skin Mucus of Yellow Catfish (Pelteobagrus fulvidraco) and Its Active Site for Chemotaxis. Int. J. Mol. Sci..

[43] Byadgi O., Chen Y.-C., Maekawa S., Wang P.-C., Chen S.-C. (2018). Immune-Related Functional Differential Gene Expression in Koi Carp (Cyprinus carpio) after Challenge with Aeromonas sobria. Int. J. Mol. Sci..

[44] Gomez D., Sunyer J.O., Salinas I. (2013). The mucosal immune system of fish: The evolution of tolerating commensals while fighting pathogens. Fish Shellfish Immunol..

[45] Ye J., Coulouris G., Zaretskaya I., Cutcutache I., Rozen S., Madden T.L. (2012). Primer-BLAST: A tool to design target-specific primers for polymerase chain reaction. BMC Bioinform..

[46] Watanuki H., Ota K., Tassakka A.C.M.A.R., Kato T., Sakai M. (2006). Immunostimulant effects of dietary Spirulina platensis on carp, Cyprinus carpio. Aquaculture.

[47] Pietsch C. (2017). Zearalenone (ZEN) and Its Influence on Regulation of Gene Expression in Carp (Cyprinus carpio L.) Liver Tissue. Toxins.

[48] Livak K.J., Schmittgen T.D. (2001). Analysis of Relative Gene Expression Data Using Real-Time Quantitative PCR and the 2−ΔΔCT Method. Methods.

[49] Dawood M.A.O., Koshio S., Esteban M.Á. (2018). Beneficial roles of feed additives as immunostimulants in aquaculture: A review. Rev. Aquac..

[50] Dawood M.A.O., Koshio S., Ishikawa M., Yokoyama S., El Basuini M.F., Hossain M.S., Nhu T.H., Moss A.S., Dossou S., Wei H. (2017). Dietary supplementation of β-glucan improves growth performance, the innate immune response and stress resistance of red sea bream, *Pagrus major*. Aquac. Nutr..

[51] Song S.K., Beck B.R., Kim D., Park J., Kim J., Kim H.D., Ringø E. (2014). Prebiotics as immunostimulants in aquaculture: A review. Fish Shellfish Immunol..

[52] Cerezuela R., Meseguer J., Esteban A. (2011). Current Knowledge in Synbiotic Use for Fish Aquaculture: A Review. J. Aquac. Res. Dev..

[53] Huynh T.-G., Shiu Y.-L., Nguyen T.-P., Truong Q.-P., Chen J.-C., Liu C.-H. (2017). Current applications, selection, and possible mechanisms of actions of synbiotics in improving the growth and health status in aquaculture: A review. Fish Shellfish Immunol..

[54] Mo F., Zhao J., Liu N., Cao L.-H., Jiang S.-X. (2014). Validation of reference genes for RT-qPCR analysis of CYP4T expression in crucian carp. Genet. Mol. Biol..

[55] McCurley A.T., Callard G.V. (2008). Characterization of housekeeping genes in zebrafish: Male-female differences and effects of tissue type, developmental stage and chemical treatment. BMC Mol. Biol..

[56] Filby A.L., Tyler C.R. (2007). Appropriate “housekeeping” genes for use in expression profiling the effects of environmental estrogens in fish. BMC Mol. Biol..

[57] Julin K., Johansen L.-H., Sommer A.-I. (2009). Reference genes evaluated for use in infectious pancreatic necrosis virus real-time RT-qPCR assay applied during different stages of an infection. J. Virol. Methods.

[58] Jorgensen S.M., Kleveland E.J., Grimholt U., Gjoen T. (2006). Validation of Reference Genes for Real-Time Polymerase Chain Reaction Studies in Atlantic Salmon. Mar. Biotechnol..

[59] Small B.C., Murdock C.A., Bilodeau-Bourgeois A.L., Peterson B.C., Waldbieser G.C. (2008). Stability of reference genes for real-time PCR analyses in channel catfish (Ictalurus punctatus) tissues under varying physiological conditions. Comp. Biochem. Physiol. Part B Biochem. Mol. Biol..

[60] Yabu T., Toda H., Shibasaki Y., Araki K., Yamashita M., Anzai H., Mano N., Masuhiro Y., Hanazawa S., Shiba H. (2011). Antiviral protection mechanisms mediated by ginbuna crucian carp interferon gamma isoforms 1 and 2 through two distinct interferon gamma-receptors. J. Biochem..

[61] Savan R., Aman A., Sakai M. (2003). Molecular cloning of G type lysozyme cDNA in common carp (Cyprinus carpio L.). Fish Shellfish Immunol..

[62] Ellis A.E. (1999). Immunity to bacteria in fish. Fish Shellfish Immunol..

[63] Hicks P.S., Saunero-Nava L., Du Clos T.W., Mold C. (1992). Serum amyloid P component binds to histones and activates the classical complement pathway. J. Immunol..

[64] Falco A., Cartwright J.R., Wiegertjes G.F., Hoole D. (2012). Molecular characterization and expression analysis of two new C-reactive protein genes from common carp (Cyprinus carpio). Dev. Comp. Immunol..

[65] Hoseinifar S.H., Ahmadi A., Raeisi M., Hoseini S.M., Khalili M., Behnampour N. (2017). Comparative study on immunomodulatory and growth enhancing effects of three prebiotics (galactooligosaccharide, fructooligosaccharide and inulin) in common carp (*Cyprinus carpio*). Aquac. Res..

[66] Hoseinifar S.H., Zoheiri F., Dadar M., Rufchaei R., Ringø E. (2016). Dietary galactooligosaccharide elicits positive effects on non-specific immune parameters and growth performance in Caspian white fish ( Rutilus frisii kutum ) fry. Fish Shellfish Immunol..

[67] Hoseinifar S.H., Sharifian M., Vesaghi M.J., Khalili M., Esteban M.Á. (2014). The effects of dietary xylooligosaccharide on mucosal parameters, intestinal microbiota and morphology and growth performance of Caspian white fish (Rutilus frisii kutum) fry. Fish Shellfish Immunol..

[68] Miandare H.K., Farvardin S., Shabani A., Hoseinifar S.H., Ramezanpour S.S. (2016). The effects of galactooligosaccharide on systemic and mucosal immune response, growth performance and appetite related gene transcript in goldfish (Carassius auratus gibelio). Fish Shellfish Immunol..

[69] Nawaz A., Bakhsh javaid A., Irshad S., Hoseinifar S.H., Xiong H. (2018). The functionality of prebiotics as immunostimulant: Evidences from trials on terrestrial and aquatic animals. Fish Shellfish Immunol..

[70] Tzortzis G., Goulas A.K., Gibson G.R. (2005). Synthesis of prebiotic galactooligosaccharides using whole cells of a novel strain, Bifidobacterium bifidum NCIMB 41171. Appl. Microbiol. Biotechnol..

[71] Vulevic J., Juric A., Tzortzis G., Gibson G.R. (2013). A mixture of trans-galactooligosaccharides reduces markers of metabolic syndrome and modulates the fecal microbiota and immune function of overweight adults. J. Nutr..

[72] Vulevic J., Drakoularakou A., Yaqoob P., Tzortzis G., Gibson G.R. (2008). Modulation of the fecal microflora profile and immune function by a novel trans-galactooligosaccharide mixture (B-GOS) in healthy elderly volunteers. Am. J. Clin. Nutr..

[73] Depeint F., Tzortzis G., Vulevic J., I’Anson K., Gibson G.R. (2008). Prebiotic evaluation of a novel galactooligosaccharide mixture produced by the enzymatic activity of Bifidobacterium bifidum NCIMB 41171, in healthy humans: A randomized, double-blind, crossover, placebo-controlled intervention study. Am. J. Clin. Nutr..

[74] Silk D.B.A., Davis A., Vulevic J., Tzortzis G., Gibson G.R. (2009). Clinical trial: The effects of a trans-galactooligosaccharide prebiotic on faecal microbiota and symptoms in irritable bowel syndrome. Aliment. Pharmacol. Ther..

[75] Drakoularakou A., Tzortzis G., Rastall R.A., Gibson G.R. (2010). A double-blind, placebo-controlled, randomized human study assessing the capacity of a novel galacto-oligosaccharide mixture in reducing travellers’ diarrhoea. Eur. J. Clin. Nutr..

[76] Vulevic J., Tzortzis G., Juric A., Gibson G.R. (2018). Effect of a prebiotic galactooligosaccharide mixture (B-GOS®) on gastrointestinal symptoms in adults selected from a general population who suffer with bloating, abdominal pain, or flatulence. Neurogastroenterol. Motil..

[77] Slawinska A., Dunislawska A., Plowiec A., Radomska M., Lachmanska J., Siwek M., Tavaniello S., Maiorano G. (2019). Modulation of microbial communities and mucosal gene expression in chicken intestines after galactooligosaccharides delivery In Ovo. PLoS ONE.

[78] Slawinska A., Plowiec A., Siwek M., Jaroszewski M., Bednarczyk M. (2016). Long-Term Transcriptomic Effects of Prebiotics and Synbiotics Delivered In Ovo in Broiler Chickens. PLoS ONE.

[79] Siwek M., Slawinska A., Stadnicka K., Bogucka J., Dunislawska A., Bednarczyk M. (2018). Prebiotics and synbiotics—In ovo delivery for improved lifespan condition in chicken. BMC Vet. Res..

[80] Slawinska A., Mendes S., Dunislawska A., Siwek M., Zampiga M., Sirri F., Meluzzi A., Tavaniello S., Maiorano G. (2019). Avian model to mitigate gut-derived immune response and oxidative stress during heat. Biosystems..

[81] Slawinska A., Zampiga M., Sirri F., Meluzzi A., Bertocchi M., Tavaniello S., Maiorano G. (2020). Impact of galactooligosaccharides delivered in ovo on mitigating negative effects of heat stress on performance and welfare of broilers. Poult. Sci..

[82] Tavaniello S., Slawinska A., Prioriello D., Petrecca V., Bertocchi M., Zampiga M., Salvatori G., Maiorano G. (2020). Effect of galactooligosaccharides delivered in ovo on meat quality traits of broiler chickens exposed to heat stress. Poult. Sci..

